# Crossing Age Boundaries: The Unifying Potential of Presepsin in Sepsis Diagnosis Across Diverse Age Groups

**DOI:** 10.3390/jcm13237038

**Published:** 2024-11-21

**Authors:** Edmilson Leal Bastos de Moura, Rinaldo Wellerson Pereira

**Affiliations:** 1 Health Sciences Doctoral Program, University of Brasília (UnB), Brasilia 70910-900, Distrito Federal, Brazil; 2 School of Health Sciences, Distrito Federal University (UnDF), Brasilia 70710-907, Distrito Federal, Brazil; 3 Genomic Sciences and Biotechnology Graduate Program, Catholic University of Brasilia, Brasilia 71966-700, Distrito Federal, Brazil

**Keywords:** sepsis, biomarkers, presepsin, elderly, neonates, adults

## Abstract

Sepsis is a pervasive condition that affects individuals of all ages, with significant social and economic consequences. The early diagnosis of sepsis is fundamental for establishing appropriate treatment and is based on warning scores and clinical characteristics, with positive microbiological cultures being the gold standard. Research has yet to identify a single biomarker to meet this diagnostic demand. Presepsin is a molecule that has the potential as a biomarker for diagnosing sepsis. In this paper, we present a narrative review of the diagnostic and prognostic performance of presepsin in different age groups. Given its particularities, it is identified that presepsin is a potential biomarker for sepsis at all stages of life.

## 1. Introduction

Sepsis is a critical clinical condition defined as life-threatening organ dysfunction secondary to a deregulated host response to infection [1]. The high prevalence of sepsis, combined with the elevated mortality rates associated with severe sepsis, imposes significant economic costs and exacts a devastating toll on human lives [2]. Sepsis impacts individuals across all age groups, considering the unique characteristics of each group.

Cardiovascular diseases and sepsis are two of the most significant contributors to human mortality [3,4]. Regarding cardiovascular diseases, heightened awareness of early signs and improvements in health services and professional training have resulted in a commendable success rate for early diagnosis [5]. Conversely, concerning sepsis, there is a lack of public understanding regarding the risks associated with infections, and healthcare professionals face challenges in identifying clear clinical signs for early diagnosis [6]. Early detection scores such as the quick Sequential Organ Failure Assessment (qSOFA) and systemic inflammatory response syndrome (SIRS) criteria and protocol adherence can facilitate the timely diagnosis and treatment of sepsis [7]. However, these scores need more inputs in their construction to ensure sensitivity and specificity for widespread use in clinical practice [8]. Identifying new biomarkers for sepsis diagnosis and prognosis becomes an essential goal in this context.

Presepsin is a biomarker with significant diagnostic and prognostic potential, making it superior to conventional biomarkers such as C-reactive protein (CRP) or procalcitonin (PCT) [9]. This analytical review aims to identify original studies in the literature that utilize presepsin as a biomarker for sepsis across all age groups, from neonates to nonagenarians, encompassing young adults and adults.

## 2. Demographics of Sepsis

Statistics from the USA indicate that sepsis was associated with 201,092 deaths in 2019 [10]. It is the primary cause of in-hospital deaths, accounting for an estimated 19.7% of the overall death rate [3]. The global incidence of sepsis exhibits a bimodal distribution, with peaks occurring in childhood and older adults [3].

Sepsis is the leading cause of death among infants and children, with an estimated 1.6 million cases per year [11], despite some variability in its occurrence, depending on the diagnostic strategy [2,12]. In the neonatal period, sepsis occurs in 1 to 5 cases per 1000 live births [13], showing an overall mortality rate of 24.4%, which can surge to as high as 54% in premature infants under 24 weeks of gestation [14].

Research indicates that >60% of sepsis cases occur in patients aged ≥65 years [15]. Mortality from sepsis in older adults constitutes approximately three-quarters of all sepsis-related deaths in the USA, particularly among individuals over the age of 65 [10]. Although this index declined between 2000 and 2019, it continues to rise with age, increasing five times in those over 85 [16].

## 3. Pathophysiology and Immunological Aspects of Sepsis Across Ages

Comprehending the pathophysiology of sepsis requires a solid understanding of the intricate interaction among various domains, precisely, the convergence of the inflammation pathway with the coagulation system, leading to endothelial stimulation and microcirculatory dysfunction [17]. This framework underpins the exploration of potential biomarkers, diagnostic approaches, optimal treatment durations, and the management of antibiotic therapy [17]. For instance, the activation and dysfunction of endothelial cells induced by sepsis tend to diminish with advanced age [18]. In this section, we delve into some age-related characteristics of the sepsis response.

Sepsis in the pediatric population is a distinct condition characterized by unique features in the host response to infection and therapy. It is crucial to understand the developmental differences that distinguish it from adult sepsis [19]. Neonatal sepsis, occurring within the first 28 days of life, presents unique aspects. Neonatal sepsis can be acquired from the mother during intrauterine life or through postpartum care [20]. Beyond the neonatal stage, the clinical signs of pediatric sepsis are nonspecific. They can be influenced by birth conditions or adaptation to extrauterine life [21], often leading to delayed diagnosis [20]. Consequently, the diagnosis is usually presumptive, and treatment is based on clinical findings and nonspecific laboratory tests.

Moreover, the definitions of sepsis currently used for this age group are an extrapolation of the criteria used for adults [22], needing more validation for pediatric patients, which results in a low predictive value [23]. This diagnostic challenge has recently been addressed to validate new pediatric sepsis and septic shock criteria through organic dysfunction variables called the Phoenix criteria [24].

The inflammatory response to sepsis in pediatric patients suggests a predominantly anti-inflammatory phenotype, which is exacerbated compared to that in adults [25]. The immaturity of the adaptive immune system causes the neonate to become more dependent on the innate immune system [26].

In children, sepsis induces an immune response characterized initially by a pro-inflammatory state, promoting classic symptomatology such as fever, tachycardia, and tachypnea [27], making it clinically indistinguishable from the inflammatory response caused by other etiologies, posing challenges for its diagnosis by pediatricians [28]. Although there is no cohesive understanding of the mechanisms involved in sepsis [29], this pro-inflammatory phase is followed by the immunoparalysis phase, characterized by anti-inflammatory activity [27]. Such information corroborates the notion that a developmental difference exists in the inflammatory response to infection or injury among children and adults, exemplified by its pattern of reduced organ failure following sepsis (and restricted systemic hyperinflammation); children may have either an attenuated proinflammatory response or an augmented antiinflammatory response [25].

The prognosis of sepsis in pediatric patients is associated with lactate clearance as well as physiological variables in the first 4 h after admission to the intensive care unit [30]. There also appears to be a correlation between genetic profiles and endotypes for septic shock in childhood [31], suggesting the possible existence of subclasses of response in sepsis. Thus, corticotherapy may benefit those subgroups [31]; however, developing clinical trials to understand immunophenotypes and their relation to immunoparalysis must improve the prognosis of childhood sepsis [27].

Scientific research on sepsis has more widely included adults. A recent study identified a relative increase in sepsis diagnoses in the 18–44 age group, possibly due to greater awareness of the syndrome in this age range [32]. In this age group, the inflammatory and immunosuppressive responses are simultaneous and exhibit interindividual variation in the conceptual model of immune trajectories before, during, and after sepsis [33]. Thus, chronic hyperinflammation and immunosuppression have a prolonged clinical trajectory, known as persistent inflammation/immunosuppression and catabolism syndrome [34,35]. This endotype leads to chronic critical illness, characterized by impaired functional status, rehabilitation failure, and increased mortality [35].

Despite the heterogeneity in biological ages among individuals of the same chronological age [36], sepsis in older adults holds significance due to its association with increased morbidity [37], positioning it as the quintessential disease affecting this demographic [36].

The immunosenescence contributes to the reduced immune response to pathogens. Additional factors related to aging, such as associated comorbidities, successive hospitalizations, institutionalization, unfavorable nutritional status, pharmacokinetic changes, polypharmacy, and the uncertain composition of the microbiome, further compound the impact on the immune system [2]. As discussed above, the low-grade and persistent pro-inflammatory state related to sepsis also contributes to immunosenescence [38].

Older age can be considered an independent predictor of mortality [15,39] despite the more encouraging results in a subgroup of patients over 85 obtained in a recent study [32], which showed a reduction in mortality (<50%) compared to previous studies [40]. As the initial signs of sepsis in this age group may be invisible, progression to septic shock can be rapid [2], highlighting the particular importance of early diagnosis in this age group.

## 4. Biomarkers and Sepsis

Biomarkers reflect the state of health or disease at a molecular level [41]. They improve diagnosis, define subsets of diseases that may differ in response, as well as individual variability in the drug’s molecular target, and provide an early reading of the response to therapy, among other functions. The search for new molecules with this purpose has been identified as a high priority for science [42] as part of the challenge of implementing “Personalized Medicine” [41].

In the case of sepsis, the question is whether it is possible to discriminate among septic patients in terms of which subgroups share specific biological characteristics, who are at risk of unfavorable outcomes, and who are at risk of organ failure [28].

Although no ideal single biomarker or even combination of biomarkers serves this purpose in the international consensuses on sepsis [1,20], their use in this context is commonplace because, besides being an important aid in diagnosis, they enable us to predict possible sepsis syndrome outcomes [43]. Unfortunately, no single one can reliably perform as a stand-alone sepsis biomarker [44,45,46,47].

Biomarkers cannot represent the uncontrolled inflammation and increased vascular permeability that characterizes sepsis, leading to hypotension and organ dysfunction [48]. Therefore, to develop rapid assessment and differentiation between infection and inflammation, biomarker research aims to enable point-of-care testing among many molecules [48]. However, new biomarkers may not present superior results to traditional ones, frustrating expectations of the benefits, suggesting their aid after evaluation with the usual scales and biomarkers [49].

Among the various functions necessary for the ideal biomarker, it should be precise for guiding therapeutic decision-making [50]. However, its measurement is impaired due to critical disadvantages, such as the collecting timing and the insufficiency of standardization (Table 1). Although traditionally measured simultaneously, gathering biomarkers at several intervals may show a better overview of the host response to sepsis [44].

Guiding therapeutic decisions should be one of the ideal features of sepsis biomarkers [50]. In this context, deriving diagnostic algorithms appears to be a reliable strategy for early diagnosis of sepsis, integrating the pretest probability of infection, clinical features, and results of in vitro diagnostic testing [70].

Considering the pathological process, the disease stage, and individual patient characteristics, a personalized therapeutic strategy could be provided by a biomarker-guided approach, avoiding “one size fits all” sepsis therapies [71]. In other words, sepsis research must consider the individual immune status or likely response to a specific treatment to avoid harmful therapy to a patient with a particular immune response activation pattern [71].

In addition to all the issues addressed so far, we must incorporate the key concept of value-based medicine, which involves cost-effectiveness studies, comparing different interventions, and defining the viability of diagnostic means. This is fundamental in a world of limited resources [46].

In pediatrics, most researchers agree that diagnostic priority depends on clinical signs and not biomarkers, even though sepsis has a polymorphic presentation [72]. CRP and PCT have been the most widely used biomarkers in pediatric clinical practice, with the recommendation that they must be used simultaneously to increase the efficiency of the results [72]. However, low accuracy is observed, as well as variable sensitivity and specificity for detecting bacterial infection via polymerase chain reaction (PCR) (lower when a single measurement is performed) [73]. Lactate is used to corroborate the diagnosis of septic shock and assess the response to therapy; however, normal or slightly elevated levels do not rule out the development of sepsis and septic shock; therefore, it is of limited effectiveness in children [74].

The medical literature comprises thousands of studies evaluating the applicability of biomarkers in adult sepsis, reporting >200 potential candidate molecules for the early diagnosis of sepsis [75]. However, methodological biases in many of these articles create limitations [76]. Due to these issues and insufficient evidence, only a few are suitable for everyday clinical use, with CRP, PCT, IL-6, and presepsin among the most promising [57]. No single biomarker has sufficient diagnostic power to be used independently; instead, a panel of biomarkers is considered the best option for a point-of-care approach to sepsis [77].

The specificity and sensitivity of biomarkers can be influenced by age. Thus, a moderate to marked increase in biomarkers such as CRP, an inflammatory peptide associated with immunosenescence, can be expected with advancing age [78]. This molecule is one of the substances linked to aging-related inflammation, and its increase is described as characteristic of the aging process [79]. In adult and older adults hospitalized with sepsis, CRP can rise within 72 h and remain elevated for extended periods in older adults, even after they are discharged from the hospital [80]. This marker has been linked to poorer clinical outcomes in these patients [81].

It seems that patients who have subclinical inflammation at the time of discharge are more likely to have a higher risk of death, as indicated by persistently elevated inflammatory biomarker levels [81]. Patients over 65 tend to have a higher baseline inflammation, as reflected in higher inflammatory biomarker levels upon admission. However, these levels converge with those found in other age groups within the first 72 h [80].

However, contrasting perspectives exist as some research groups have yet to identify a robust association between aging and markers of systemic inflammation or cytokine release in sepsis [18]. Furthermore, older adults experiencing sepsis display a dampening of endothelial cell activation, termed endothelial tolerance. Significantly, this phenomenon is attributed to the septic event rather than age [18].

## 5. Presepsin as a Sepsis Biomarker Across Age Groups

Presepsin is a molecule identified in many cells involved in the sepsis cascades, including macrophages, monocytes, and granulocytes, and is responsible for the intracellular transduction of endotoxin signals [82]. This fragment of sCD14 (soluble glycoprotein receptor) is highly correlated with bacterial infection [67], directly linked to phagocytosis resulting from this process [59]. There are no reports of increasing presepsin levels in patients with viral infections [60], so cutoff levels are used to distinguish from nonbacterial infectious diseases, as long as PSP is present in very low concentrations in the serum of healthy individuals [61]. Thus, the biomarker PSP supports the paradigm that surrogates of innate antimicrobial defense could aid in the diagnosis of sepsis [64].

Granulocytes phagocytize bacteria and CD14 and secrete presepsin into the blood within 2 h after enzymatic digestion [83]. During the induction of systemic inflammation, the increase in presepsin levels occurs earlier and more rapidly than other sepsis markers [82].

Presepsin has advantages that justify its use, such as its early elevation in infection [84], high accuracy [20], and affordability compared to the gold standard blood culture test (USD 7 versus USD 11–89) [85,86]. It also exhibits better prognostic validity than the PCT, CRP, and erythrocyte sedimentation rate (ESR) [87,88]. Presepsin’s advantages can be explained by its correlation with the sepsis pathophysiology, unlike other biomarkers resulting from a general inflammatory reaction [89].

**Figure 1:**
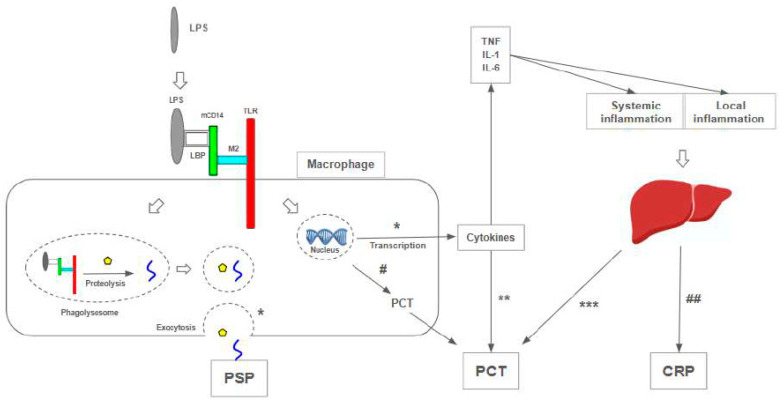
(*) The molecular complex LPS-LBP-mCD14-M2-TLR is internalized into a phagolysosome; proteolysis and internalization processes release presepsin (PSP), released in circulation after exocytosis. CD 14 promotes the expression of genes responsible for the immune response, such as cytokine production [95]. (**) TNF, IL-1, IL-2, and IL-6 levels increase PCT [96]. (***) The liver is considered the most important site to produce PCT during an inflammatory response, especially when induced by bacterial infections [97]. (#) Peripheral blood mononuclear cells express PCT on mRNA and protein levels [98]. (##) CRP is an acute-phase protein, and its synthesis is rapidly upregulated, principally in hepatocytes, under the control of cytokines [99]. LPS: lipopolysaccharide, TLR: Toll-like receptor; LBP: Lipoprotein Binding Protein; mCD14: membrane-bound CD14; M2: co-protein of TLR; TNF: tumor necrosis factor; IL-1: interleucin-1; IL-6: interleucin-6; CPR: C-reactive protein; PCT: procalcitonin; PSP: presepsin.

Although it showed inferior performance to PCT as a predictor of bacterial infection [74] (Figure 1), it showed comparable sensitivity and specificity values to those of PCT during bacterial sepsis, along with a decline in response to appropriate therapy, making it the most promising among emerging biomarkers [90]. Some authors have demonstrated better results of PSP compared to PCT in predicting mortality of sepsis at 30 days [91], also showing the prognostic value of PSP as an independent risk factor for 30-day mortality [92]. This prognostic value was demonstrated not only in single measures [93,94], but also based on dynamic monitoring [9,63].

However, PSP exhibits some particularities in individuals with impaired renal function that require caution in interpreting its values. Since it is presumably reabsorbed and catabolized in the proximal tubular cells [100], the decrease in renal function influences its diagnostic accuracy [101]. Additionally, increases in presepsin levels in hemodialysis (HD) patients may be related to the activation of neutrophils and/or monocytes, leading to presepsin release from monocytes [102]; however, it could be removed from the circulation using different modalities of renal replacement therapy (RRT), therefore, potentially causing falsely low PSP levels [101].

Although the use of isolated biomarkers presents limitations for the early diagnosis of sepsis, the combination of the two is reported to have greater specificity when compared to using PCT or PSP alone [103]. Thereby, the use of multi-marker panels may also be a promising approach [44].

The availability of laboratory assays that can measure presepsin in 17 min is another factor that has made it a promising marker in sepsis [58,82,90,95,104,105,106]. However, its use has disadvantages, such as non-standardized cutoff points and the fact that it is inaccessible in most clinical settings [20]. As discussed below, its use as a biomarker should be customized according to the age group, as the threshold values can vary.

### 5.1. Presepsin as a Sepsis Biomarker in Neonates and Children

Due to its superior diagnostic performance compared to PCT and CRP [107], presepsin use has been highlighted in neonatal sepsis. Among healthy neonates, presepsin has an average plasmatic value of 649 ng/L and 720 ng/L in premature infants [52] (Table 1). A cutoff point of 788 ng/L, 93% sensitivity, and 100% specificity was obtained to diagnose early sepsis in premature infants [53] (Table 1). Its use is advocated for monitoring antibiotic therapy, as its levels decrease when treatment is effective [108]. In neonates with infection, it has the advantage that its levels are not influenced by gestational age or other perinatal factors [95]. High serum values also increase 30-day mortality [107].

Despite having demonstrated good accuracy in several studies, using PSP as a tool in diagnosing and prognosis neonatal sepsis still requires refinement. The differentiation of biomarker behavior can be made between term and preterm neonates [52,53] and between early onset (in the first 72 h of life) and late onset [20,53,108], among others. In this age group, the diagnostic process must be remarkably rapid because, in addition to threatening life, it is a potential cause of permanent sequelae in survivors [20]. Therefore, some answers are necessary to consolidate the role of PSP as a biomarker in newborns, especially the diagnostic cutoff values, a topic that is still controversial (Table 1). Celerity, sensitivity, and specificity would reduce unnecessary treatments in symptomatic low-risk individuals.

In children, presepsin shows similar responses; in a recent meta-analysis, presepsin showed high sensitivity and diagnostic accuracy compared to PCR and PCT but lower specificity [100]. The usefulness of presepsin extends to individuals with hematological neoplasms, where it can be a good predictor of clinical evolution with septic shock in febrile neutropenics [101]. In these patients, when there is no detectable site of infection, higher levels of presepsin can anticipate the positive result of cultures, discriminating the infectious origin of the febrile condition [56,109] (Table 2).

Biomarkers in pediatric sepsis are a valuable aid in promptly and cautiously diagnosing sepsis. Despite the emergence of promising options, such as genomic biosignature [29], older biomarkers, including CRP, PCT, ferritin, and lactate, despite their varying levels of reliability, continue to serve as useful clinical adjuncts in diagnosis [29]. Moreover, they are more readily available in most pediatric institutions [29].

Additionally, laboratory tests can determine the severity of sepsis, such as quantifying dynamic changes in levels of the antigen-presenting molecule human leukocyte antigen-DR isotype or the production of TNF-α upon stimulation (the latter representing the hyporeactivity of the innate immune system) [27].

### 5.2. Presepsin as a Sepsis Biomarker in Adults

As discussed previously, many current studies focus on adults, who benefit most from the results validated by the scientific literature.

With average plasmatic levels of 202 pg/mL in healthy individuals [61], the increase in presepsin levels in the bloodstream correlates with the pathophysiology of sepsis rather than a general inflammatory reaction [89]. This characteristic gives it better prognostic validity than PCT, CRP, and ESR [87,88].

Presepsin levels have been shown to correlate with the severity and in-hospital mortality of patients with sepsis and septic shock [9], with mean values of 1718 and 3266 pg/mL for survivors and nonsurvivors, respectively [66] (Table 1). In a 28-day survival period, significant values of 1108 vs. 3251 pg/mL were obtained for survivors and nonsurvivors, respectively [65] (Table 1). Blood level changes, both absolute (increase above 500 pg/L) [110] and relative (reduction of >50% between admission and the seventh day) [111], correlated with unfavorable and favorable clinical outcomes, respectively. Due to its stability in various acute or chronic clinical scenarios, presepsin has helped detect sepsis in liver cirrhosis [112], rheumatoid arthritis [113], and febrile neutropenia [114], among others.

However, presepsin showed an inferior performance than PCT as a predictor of bacterial infection and bacteremia, proven by culture [88]. Furthermore, it requires adjustments as a biomarker in patients with altered kidney function [67,115] (Table 1 and Table 2).

### 5.3. Presepsin as a Sepsis Biomarker in Older Adults

Studies suggest that presepsin could be more valuable than PCT and CRP as a predictor of bacteremia in older adult patients admitted to the emergency department. It showed significantly higher values than those without bacteremia (866.6 ± 184.6 vs. 639.9 ± 137.1 ng/L, *p* = 0.03) [112] (Table 1). It showed similar diagnostic and prognostic accuracy to PCT and early warning scores (qSOFA and SIRS), with the combination of the three biomarkers being superior to the use of anyone alone [69].

Aging was found to be an independent predictor of increased blood presepsin levels (Claessens 2017), with a significant difference comparing over 70 and under-70 age groups 470 (380–601) ng/L vs. 300 (201–457) ng/L, *p* < 0.001) [61]. Notably, age-related changes in renal and vascular function, such as glomerulosclerosis, vascular dysautonomia, altered tubular management of creatinine, and reduced renal reserve [114], increase presepsin levels in renal dysfunction [116,117,118]. A study revealed that in older adult patients, hypercreatinemia raises the presepsin threshold value to 706 ng/L, enabling a diagnosis of sepsis [119].

However, there are caveats in the literature. Some authors postulate that for individuals over 75 years of age, a cutoff point of 380 pg/mL would be more appropriate [113]. This differs from the findings of systematic reviews focusing on predominantly younger populations, in which levels as high as 600 ng/L were found [59,60]. The rationale for this lies in the origin of presepsin; it comes from granulocytes, which are dysfunctional in this age group and hyporesponsive to infectious stimuli [69], a characteristic of immunosenescence (Table 2).

## 6. Published Meta-Analysis on Presepsin as a Sepsis Biomarker

Published meta-analyses corroborate the promising role of presepsin as a biomarker in sepsis. In a search covering the period from 2010 to the present, several meta-analyses were found on using presepsin in neonatal sepsis. This search evaluated 28 studies and 2505 patients, recognizing the diagnostic value of presepsin in early-onset sepsis (i.e., occurring in the first 72 h of life) [20] and late-onset sepsis [57,120] (Table 3).

The meta-analyses involving adults and older adults, evaluating the efficacy of presepsin in the context of sepsis, showed six meta-analyses in a literature review covering the period from 2010 to the present. It covered 20,544 patients in 141 selected studies, which, in general, showed good or moderate diagnostic accuracy in differentiating septic and nonseptic patients [121,122,123], indicating its suitability as a biomarker similar to PCT in the early diagnosis of sepsis [124] and showing relevant prognostic value [125,126] (Table 3). None of these meta-analyses categorized older adult patients into subgroups, with mean ages ranging from 55.2 years [62] to 74 years [64], demanding efforts to conduct this type of study on the older adult population or to analyze a subgroup of this age group to support the understanding of sepsis in this population.

## 7. Discussion

The pathophysiological complexity of sepsis is acknowledged as the primary impediment to developing validated biomarkers, with current emphasis on the extensive study of inflammatory markers [17]. Nevertheless, distinct age groups manifest unique characteristics in their immune responses, encompassing both pro- and anti-inflammatory aspects and phenomena like immunoparalysis and immunosenescence. This variation aligns with differences in clinical and laboratory presentations, particularly concerning the levels of inflammatory biomarkers.

Prioritizing the characterization of septic syndrome behavior across different age groups affected by it is essential. Gaining insights into this aspect and recognizing the relative significance of biomarkers can aid in developing reproducible tools. These tools, in turn, facilitate the translation of clinical research findings into practical applications at the bedside.

The clinical and laboratory characteristics of different age groups present diagnostic challenges. In newborns, biomarkers show great potential for improving diagnosis, and blood cultures, though considered the gold standard, have limitations. Blood cultures typically require a long turnaround time, ranging from 6 h to 5 days for microorganisms to reach detectable levels, with an additional 24–48 h needed for antibiotic susceptibility testing [50]. However, in newborns, the sensitivity of blood cultures is often reduced due to factors such as low blood volume during collection, low or intermittent bacteremia [107], and maternal antibiotic therapy, all of which can contribute to false-negative results [108]. Additionally, biomarkers provide insights into the newborn’s response to therapeutic interventions [13,51], thus potentially reducing the indiscriminate use of antibiotics.

Similarly, PSP has shown diagnostic and prognostic value in adult studies, where we found the most significant number of publications. Consequently, its absolute plasma values and dynamic changes [63] have been described in various clinical situations, whether associated with sepsis or not. This predominance, combined with the intrinsic diagnostic difficulty of septic syndrome, strengthens the prominence gained by PSP as an adjunct in propaedeutics.

Despite comprising a substantial proportion of intensive care unit (ICU) patients, older adults must be more adequately represented in clinical trials, hindering the development of targeted protocols [36]. This underrepresentation can be attributed to age discrimination and significantly influences the formulation of public health policies for conditions such as sepsis.

Nevertheless, older adult survivors of intensive care frequently encounter sequelae and an accelerated age-related functional decline [127]. This scenario underscores the imperative for heightened support post-hospital discharge, particularly evident in 37.3% of patients over 85 years [32]. This population’s unique characteristics and specific needs emphasize the requirement for enhanced scientific rigor in studies encompassing this demographic. Focused clinical research on this cohort would yield invaluable insights for clinical decision-making, highlighting the importance of utilizing biomarkers to inform and streamline the process.

We observe a growing endorsement for personalized medicine, extending beyond ethnic groups to encompass individualized treatment strategies. The rationale for tailored therapies is firmly grounded in robust theoretical frameworks. Early diagnosis is pivotal in accelerating the protracted and time-intensive propaedeutic process. Hence, it becomes imperative to streamline the diagnostic trajectory of sepsis by seamlessly integrating clinical and laboratory data. This integration facilitates the anticipation of therapeutic decisions and interventions, mitigates potential complications, and optimizes overall outcomes. In pursuing a personalized, pragmatic, and efficient approach to sepsis, utilizing a multi-biomarker model propelled by genomic tools holds promise for future disease management [11].

A promising trajectory for the future of sepsis management may lie in “omics” approaches, encompassing genomics, proteomics, metabolomics, and transcriptomics, alongside noteworthy strides in therapeutic interventions to optimize outcomes [128]. An illustrative instance involves the application of transcriptomic analysis panels to blood samples, enabling a precision-oriented approach in administering antimicrobials for the targeted exclusion of bacterial infections [129].

While there is a growing global awareness of sepsis, this heightened recognition has yet to translate into a substantive improvement in its management, particularly in developing or low-income countries [130]. Vulnerable populations in these regions necessitate tailored strategies, given the unavailability or unaffordability of expertise and technologies [130]. Biomarkers, emerging as promising tools, offer potential alternatives to facilitate decision-making and should be integrated into health policies.

## 8. Conclusions

Due to its unique characteristics, presepsin stands out as a promising biomarker for the diagnosis, therapeutic monitoring, and prognosis of sepsis across all age groups. Incorporating presepsin into quality improvement programs and consensus guidelines hinges on the foundation of rigorous research that validates its efficacy and solidifies its routine application.

## Figures and Tables

**Table 1:** Admission levels of presepsin—comparison between sepsis and non sepsis, and suvivor and non survivors. Cutoff values of presepsin in all stages of life.

Age Group	Author	Number of Patients (n)	Septic Group Mortality—% (Period in Days)	Admission Medium PSP Levels (ng/mL)				Cutoff Values (ng/mL)
				Sepsis	Non sepsis	Survivor	Non Survivor	
Neonates & children	Poggi et al. 2015 [51]	40	21	1295	562	-	-	885
	Pugni et al. 2015 [52]	684	-	-	649	-	-	-
	Montaldo et al. 2016 [53]	70	9	598	328	-	-	788 *
	Korpelainen et al. 2017 [54]	87	3 (in-hospital)	1432	-	-	-	-
	Bellos et al. 2018 [55]	783	-	-	-	-	-	650–850 **
	Baraka et al. 2018 [56]	60	-	1014	178	-	-	Multiple
	Yoon et al. 2019 [57]	308	-	-	-	-	-	650 **
	Puspaningtyas et al. 2023 [58]	100	5,6	806.5	717	-	-	761 *
Adults	Shozushima et al. 2011 [59]	169	-	817.9	190	-	-	399
	Endo et al. 2012 [60]	185	-	1579	312	-	-	Multiple
	Giavarina et al. 2015 [61]	200	-	55–184	-	-	-	-
	Ali et al. 2016 [62]	76	57.6 (28)	1183	472	615.5	1301	Multiple
	Yu et al. 2017 [63]	109	59.6 (90)	-	-	1230.5	1269	-
	Claessens et al. 2017 [64]	359	-	476	200	-	-	-
	Ikeda et al. 2019 [65]	129	22.5 (28)	-	-	3251	1108	-
	Zvyagyn et al. 2019 [66]	41		-	-	1718	3266	-
	Dragoş et al. 2023 [67]	510	45	1039	372	-	-	-
Old adults	Imai et al. 2019 [68]	46	-	639.93	866.56	-	-	285
	Ruangsomboon et al. 2020 [69]	250	48,2 (30)	746	316	470	795	Multiple

* Best of multiple values; ** Best accuracy values in the metanalysis.

**Table 2:** Positive and negative aspects of presepsin in all stages of life.

Aspects	Pediatric	Adult	Elderly
Positive	Early elevation, affordable cost, better diagnostic performance (PCT and CRP) and prognostic validity (30-day mortality), monitoring of antibiotic therapy, levels not influenced by gestational age, predictor of clinical evolution in febrile neutropenics	Better prognostic validity (PCT, CRP, ESR), correlation with hospital mortality in sepsis and septic shock, prognostic validity (28-day mortality), correlation with clinical outcomes, stable in different clinical scenarios (cirrhosis, rheumatoid arthritis, febrile neutropenia)	A better predictor of bacteremia in the Emergency Department (PCT, CRP), similar diagnostic accuracy to PCT, similar prognostic accuracy (qSOFA, SIRS)
Negative	Poor predictor of bacterial infection (PCT), non-standardized cutoff points, inaccessible in most scenarios	Poor predictor of bacterial infection (PCT), requires adjustments when kidney function is altered	Diagnostic and prognostic accuracy lower than combination (PCT + CRP + PSP), major renal dysfunction in older adults, specific cutoff point (immunosenescence)

PSP: presepsin; CRP: C-reactive protein; PCT: procalcitonin; ESR: erythrocyte sedimentation rate; qSOFA: quick Sequential Organ Failure Assessment; SIRS: systemic inflammation response syndrome.

**Table 3:** Meta analysis on presepsin main results.

Period		Authors	Studies Included (Number of Patients, n)	Main Conclusions
Neonatal	Early-onset	Hincu et al., 2020 [20]	28	PSP increases in the first 24 h; not influenced by GA, postnatal age or by other perinatal factors; monitoring the response to therapy; high accuracy.
	Late-onset	Yoon et al., 2019 [57]	4 (308)	PSP showed higher sensitivity and accuracy but relatively lower specificity for the diagnosis of pediatric sepsis than either PCT or CRP.
		Maldeghem et al., 2019 [120]	10 (1369)	PSP is a promising diagnostic biomarker for EOS and LOS.
Adults		Wu et al., 2017 [121]	9 (2159)	PSP is a promising marker for diagnosis of sepsis as PCT or CRP; it cannot be recommended as a single test for sepsis diagnosis.
		Zheng et al., 2015 [122]	8 (1757)	PSP has moderate diagnostic capacity for the detection of sepsis.
		Liu et al., 2016 [123]	86 (10,438)	PSP has moderate diagnostic utility in differentiating sepsis from SIRS.
		Kondo et al., 2019 [124]	19 (3012)	PSP has similar diagnostic accuracy to PCT in detecting infection, and is useful for early diagnosis of sepsis and subsequent reduction of mortality.
		Yang et al., 2018 [125]	10 (1617)	PSP first day levels had prognostic value to predict mortality in adult patients with sepsis, especially in-hospital or 30-day mortality.
		Zhu et al., 2019 [126]	9 (1561)	PSP is a promising biomarker for the prognosis of mortality in sepsis.

PSP: presepsin; GA: gestational age; EOS: early-onset sepsis; LOS: late-onset sepsis; PCT: procalcitonin; CRP: C-reactive protein.
